# Homing of Regulatory T Cells to Human Skin Is Important for the Prevention of Alloimmune-Mediated Pathology in an In Vivo Cellular Therapy Model

**DOI:** 10.1371/journal.pone.0053331

**Published:** 2012-12-31

**Authors:** Fadi Issa, Joanna Hester, Kate Milward, Kathryn J. Wood

**Affiliations:** Transplantation Research Immunology Group, Nuffield Department of Surgical Sciences, University of Oxford; MRC National Institute for Medical Research, United Kingdom

## Abstract

Regulatory T cell (Treg) therapy for immune modulation is a promising therapeutic strategy for the treatment and prevention of autoimmune disease and graft-versus-host disease (GvHD) after bone marrow transplantation. However, Treg are heterogeneous and express a variety of chemokine receptor molecules. The optimal subpopulation of Treg for therapeutic use may vary according to the pathological target. Indeed, clinical trials of Treg for the prevention of GvHD where the skin is a major target of the anti-host response have employed Treg derived from a variety of different sources. We postulated that for the effective treatment of GvHD-related skin pathology, Treg must be able to migrate to skin in order to regulate local alloimmune responses efficiently. To test the hypothesis that different populations of Treg display distinct efficacy *in vivo* based on their expression of tissue-specific homing molecules, we evaluated the activity of human Treg derived from two disparate sources in a model of human skin transplantation. Treg were derived from adult blood or cord blood and expanded *in vitro*. While Treg from both sources displayed similar *in vitro* suppressive efficacy, they exhibited marked differences in the expression of skin homing molecules. Importantly, only adult-derived Treg were able to prevent alloimmune-mediated human skin destruction *in vivo*, by virtue of their improved migration to skin. The presence of Treg within the skin was sufficient to prevent its alloimmune-mediated destruction. Additionally, Treg expressing the skin homing cutaneous lymphocyte antigen (CLA) were more efficient at preventing skin destruction than their CLA-deficient counterparts. Our findings highlight the importance of the careful selection of an effective subpopulation of Treg for clinical use according to the pathology of interest.

## Introduction

Regulatory T cells (Treg) are a population of FOXP3-expressing CD4^+^ T cells that play a central role in maintenance of self-tolerance and immune homeostasis. Treg can be divided into two populations: thymus-derived, naturally occurring nTreg and adaptive aTreg, induced after antigen exposure from conventional CD4^+^ T cells. Both umbilical cord blood (UCB) and adult peripheral blood (APB) contain functional Treg, which may be expanded *in vitro* without loss of suppressive capacities [1]. Human FOXP3-expressing CD4^+^ T cells may be divided into FOXP3^lo^CD45RA^+^ resting Treg cells, FOXP3^hi^CD45RA^−^ activated Treg and FOXP3^lo^CD45RA^−^ cytokine-producing non-Treg cells [2]. UCB-derived Treg consist mainly of naïve CD45RA^+^ cells whereas APB-derived Treg are a mixture of naïve CD45RA^+^ and memory CD45RO-expressing cells [3].

Immunomodulation with Treg is a promising therapy for autoimmune disease and for improving allograft survival after cell or solid organ transplantation. Treg have demonstrated efficacy in pre-clinical models [4], [5], with both UCB-derived and APB-derived nTreg therapy progressing to phase 0/I clinical trials for the prevention of graft-versus-host disease (GvHD) after bone marrow transplantation (BMT) [6], [7]. However nTreg are not a homogeneous population, as different subpopulations of Treg express distinct levels of functional molecules. Dissimilarities are particularly evident in the expression of tissue-specific homing receptors and molecules associated with a memory phenotype [2], [8], [9], [10], [11], [12]. Treg act both systemically and locally, with the local effects being critical for their immunoregulatory capabilities [8], [11], [13], [14], [15]. For the treatment of GvHD-related skin pathology, we postulated that Treg must be able to migrate to skin in order to regulate local alloimmune responses efficiently. To test the hypothesis that different populations of Treg display distinct efficacy *in vivo* based on their expression of tissue-specific homing molecules, we evaluated the activity of human Treg derived from two disparate sources in a model of human skin transplantation [16].

## Results and Discussion

### Allograft-infiltrating Human Treg Protect Human Skin from Destruction by Allogeneic Peripheral Blood Mononuclear Cells (PBMC)

**Figure 1:**
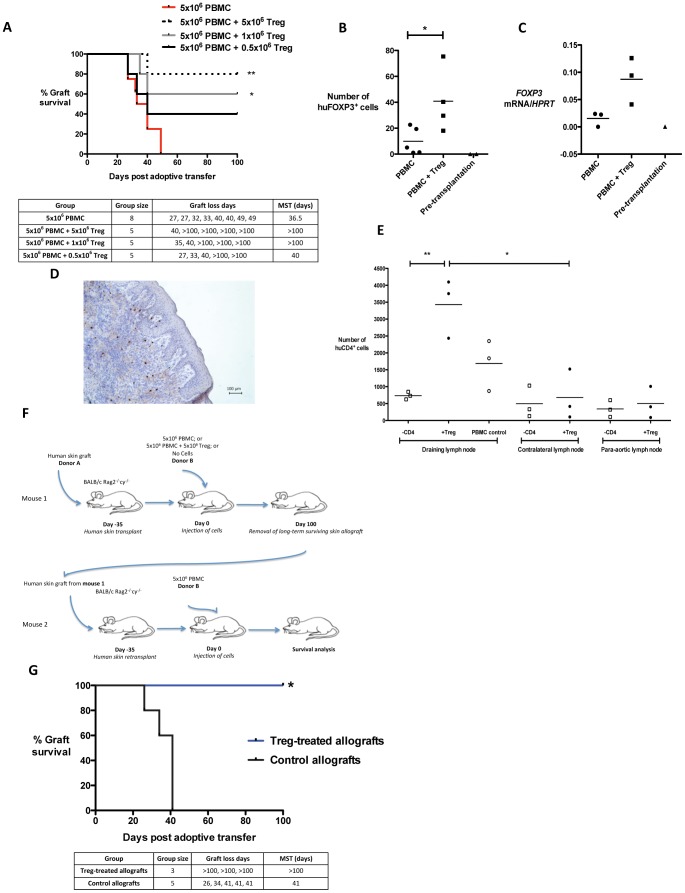
Therapeutic human Treg migrate to the human skin allograft and its draining lymph node to prevent skin destruction. (**A**) Therapy with *ex vivo*-expanded human Treg promotes the long-term survival of human skin allografts in a dose-dependent manner (**p = 0.0061; *p = 0.0386, median survival time = MST). In human skin allografts from Treg-treated mice at day 40 post-adoptive transfer of cells, an increase in the number of human CD4^+^FOXP3^+^ (huFOXP3^+^) cells is present in the skin allograft, as measured by (**B**) immunohistochemistry and (**C**) qPCR for *FOXP3*. (**D**) HuFOXP3^+^ cells are visualised in regulated human skin allografts over 100 days post-adoptive cellular transfer. (**E**) Mice received a human skin allograft and an injection of CD4^+^-depleted PBMC (-CD4^+^ group), CD4^+^-depleted PBMC with human Treg (+Treg group), or unmanipulated PBMC alone. In this system where the only human CD4^+^ (huCD4^+^) cells present are Treg, huCD4^+^ cells accumulate in the skin allograft draining lymph node at day 21 post-adoptive transfer in mice receiving Treg (n = 3 mice per group, **p = 0.0062, *p = 0.0144, data represented as values and calculated means). (**F**) Schematic representation of the experimental plan for (**G**), where mice received a human skin graft and an injection of PBMC, PBMC with Treg, or no cells. Mice receiving PBMC alone rejected their skin allografts. Skin grafts on mice receiving PBMC with Treg (‘Treg-treated allografts’) or no cells (‘Control allografts’) survived long-term. These skin grafts were retransplanted onto mice that were then reconstituted with PBMC. Skin re-transplants from Treg-treated mice survived long-term (*p* = 0.0169).

We treated humanised BALB/c Rag2^−/−^cγ^−/−^ mice that were transplanted with a human skin allograft with *ex vivo*-expanded human Treg derived from healthy adult donors. Treatment with Treg robustly extended the survival of human skin allografts in a dose-dependent manner (**Figure 1A**). 40 days after the adoptive transfer of cells, a significant number of intragraft human FOXP3^+^ cells together with increased intragraft *FOXP3* gene expression was detected in human skin grafts from mice treated with Treg (**Figures 1B and 1C**). These intragraft FOXP3^+^ cells persisted beyond 100 days within long-term surviving skin grafts (**Figure 1D**). Detection of human FOXP3^+^ cells in skin-draining lymph nodes by flow cytometry proved challenging due to their relatively low frequency. To overcome this challenge, BALB/c Rag2^−/−^cγ^−/−^ mice were transplanted with human skin and reconstituted with CD4^+^-depleted PBMC with or without an additional inoculation of Treg, and the skin-draining and contralateral axillary lymph nodes analysed by flow cytometry for the presence of human CD4^+^ cells. In this system, where the only human CD4^+^ cells present are Treg, CD4^+^ cells were found to preferentially home to the skin-draining lymph nodes (**Figure 1E**). To confirm the functionality of the FOXP3^+^ human cells within the skin allograft, long-term surviving human skin allografts from Treg-treated mice or mice not receiving human cells were retransplanted onto mice that were then reconstituted with fresh PBMC (**Figure 1F**). Skin re-transplants taken from Treg-treated mice survived long-term whereas re-transplants taken from mice not receiving human cells were rejected with normal kinetics (**Figure 1G**). In summary, therapeutic Treg home to and infiltrate human skin allografts in sufficient numbers to control alloresponses locally to prevent graft destruction.

### Ex Vivo-expanded Human Treg Derived from APB or UCB Display Different Skin-Homing Molecule Expression Patterns but Similar in vitro Suppressive Potency

**Figure 2:**
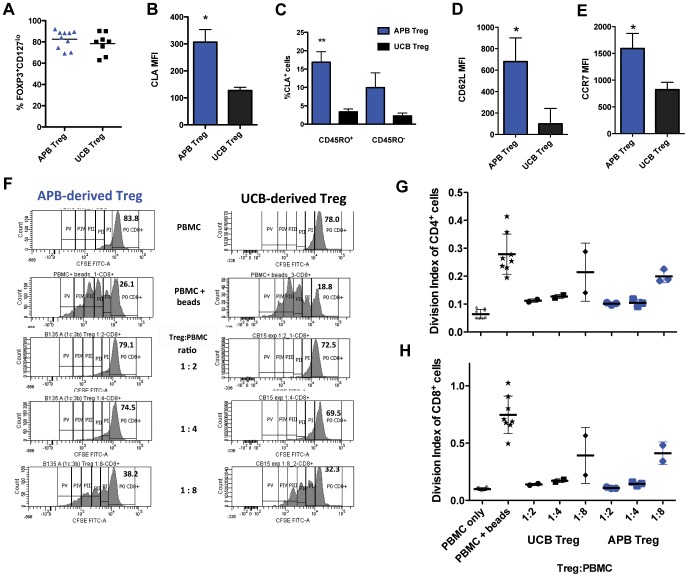
*Ex vivo*-expanded APB-derived and UCB-derived Treg express similar levels of FOXP3 and are equally suppressive *in vitro*, but express dissimilar levels of skin-homing and lymphoid-homing molecules. (**A**) After *ex vivo*-expansion, APB and UCB-derived Treg contain a similar proportion of FOXP3^+^CD127^lo^ cells. Each data point corresponds to a distinct donor. (**B**) Expression of CLA is significantly higher in APB-derived Treg (*p* = 0.0254). (**C**) The higher expression of CLA is related to a higher frequency of CD45RO^+^ cells in APB-derived Treg (*p = 0.0116). The expression of the lymphoid-homing receptors (**D**) CD62L and (**E**) CCR7 is higher in adult blood-derived Treg compared with UCB-derived Treg (*p* = 0.0191 and *p* = 0.0133 respectively). Data are represented as mean +/− standard deviation (SD). (**F**) CFSE-labelled CD8^+^ PBMC responders stimulated with αCD3/αCD28 beads are suppressed to a similar degree by APB-derived and UCB-derived Treg. Figures indicate the percentage of undivided CD8^+^ responder cells. *In vitro* suppression efficacy expressed as division index of both (**G**) CD4^+^ and (**H**) CD8^+^ responders is similar for both APB-derived and UCB-derived Treg. Data are represented as individual data points with calculated means.

Therapeutic Treg that have been trialled clinically for the prevention of GvHD have been derived and expanded *in vitro* from either APB or UCB [6], [7]. However, Treg are not necessarily a homogeneous population and functional changes in Treg may occur with aging [2], [17]. We expanded human Treg from APB and UCB and analysed their expression of FOXP3, CD127, cutaneous lymphocyte antigen (CLA), CD62L and CCR7. CLA is a key homing molecule for the migration of leukocytes to skin [9], [18]. Expanded populations of APB-derived and UCB-derived Treg contained similar proportions of FOXP3^+^CD127^lo^ cells (**Figure 2A**). In contrast, APB-derived Treg displayed a higher CLA mean fluorescence intensity (MFI) than UCB-derived Treg (**Figure 2B**). APB is known to contain a higher frequency of CD45RO^+^ Treg than UCB [17]. Interestingly, it is this CD45RO^+^ fraction of cells that contained the highest frequency of CLA positivity (**Figure 2C**). Of note, we tested the expression of other skin homing receptors including CCR4 and CCR6 but found no difference in the levels of expression between APB and UCB-derived Treg (*data not shown*). APB-derived Treg also displayed a higher MFI for the lymphoid homing receptors CD62L and CCR7 (**Figures 2D and 2E**). Expression of FOXP3 is critical for the regulatory activity of Treg [19]. As APB-derived and UCB-derived Treg contain a similar frequency of FOXP3^+^ cells, it would therefore follow that the suppressive efficacy *in vitro* would be similar for both populations. Indeed, in a CFSE proliferation assay, both populations of Treg suppressed the proliferation of non-autologous CD8^+^ and CD4^+^ responders to a similar degree (**Figures 2F, 2G and 2H**).

### APB-derived Treg are More Effective at Suppressing Alloresponses Against Human Skin in vivo

**Figure 3:**
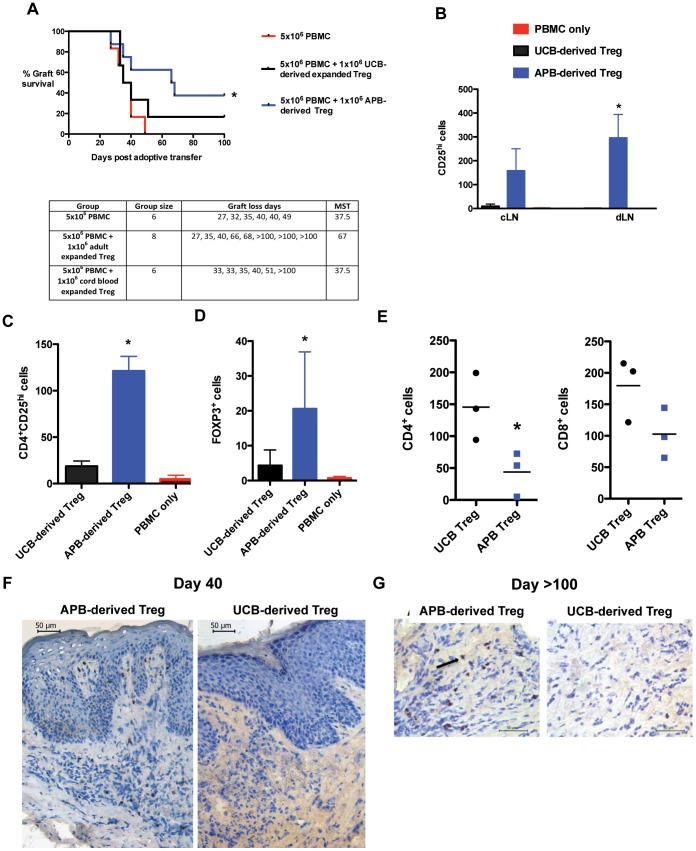
Only APB-derived Treg are effective at prolonging human skin allograft survival *in vivo*. (**A**) PBMC-reconstituted mice transplanted with human skin allografts were treated with either APB-derived or UCB-derived non-autologous *ex vivo*-expanded human Treg at a 5∶1 ratio of PBMC to Treg. Prolonged survival (MST 67 days *cf.* 37.5 days) was only achieved with APB-derived Treg (*p* = 0.0323). (**B**) Flow cytometric analysis of lymph nodes from human skin-transplanted mice at day 40 post-adoptive transfer of cells demonstrating a higher number of CD4^+^CD25^hi^ cells in the skin-draining lymph node (dLN, *p* = 0.0398) and a similar trend in the contralateral lymph node (cLN) in mice treated with APB but not UCB-derived Treg. Data are represented as mean +/− SD. (**C**) Similarly, in the skin graft a higher number of CD4^+^CD25^hi^ cells was detected by flow cytometry in mice treated with APB-derived compared with UCB-derived Treg (*p* = 0.0154). (**D**) A significantly higher number of human FOXP3^+^ cells was detected by immunohistochemistry in skin allografts from mice treated with APB-derived Treg compared with UCB-derived Treg (examined at day 35–40, *p* = 0.0273, data are represented as mean +/− SD). (**E**) At day 40, a reduced number of skin infiltrating CD4^+^ (p<0.05) and CD8^+^ (not significant) T cells was detected by immunohistochemistry in skin allografts from mice treated APB-derived Treg compared with UCB-derived Treg. (**F**) At day 40, human FOXP3^+^ cells are visible throughout the dermis and epidermis of human skin grafts from APB-derived Treg-treated mice but not UCB-derived Treg-treated mice. (**G**) At day 100, human FOXP3^+^ cells remain visible throughout human skin grafts from mice treated with APB-derived but not UCB-derived Treg.

To determine whether APB-derived Treg are more effective *in vivo* by virtue of their expression of skin-homing molecules, we reconstituted human skin-transplanted BALB/c Rag2^−/−^cγ^−/−^ mice with allogeneic PBMC and treated separate groups of mice with non-autologous Treg derived from either APB or UCB. Only Treg derived from APB were effective at significantly prolonging the survival of the skin transplant, achieving a median survival time (MST) of 67 days compared with 37.5 days using UCB-derived Treg or PBMC alone (**Figure 3A**). At day 40 post-adoptive transfer of cells, a significantly higher number of human CD4^+^CD25^hi^ cells was detected in the skin-draining axillary lymph node of mice treated with APB-derived Treg compared with UCB-derived Treg (**Figure 3B**). A similar trend was observed in the contralateral lymph node, indicating that APB-derived Treg have a higher propensity to migrate to lymphoid tissue and in particular to the allograft-draining lymph node. Moreover, a higher number of human CD4^+^CD25^hi^ and FOXP3^+^ cells was detected in the skin graft by flow cytometry and immunohistochemistry in mice treated with APB-derived Treg compared to UCB-derived Treg (**Figures 3C and 3D**). At day 40 after adoptive transfer, the number of allograft-infiltrating CD4^+^ and CD8^+^ T cells was reduced in mice treated with APB-derived Treg as compared to those treated with UCB-derived Treg (**Figure 3E**). Importantly, in mice treated with APB-derived Treg, FOXP3^+^ cells could be visualised throughout the skin transplant at day 40 post-adoptive transfer (**Figure 3F**) and even remained detectable beyond 100 days (**Figure 3G**).

### CLA^+^ Treg Home to the Skin and are More Effective at Preventing Skin Allograft Destruction than CLA^−^ Treg

**Figure 4:**
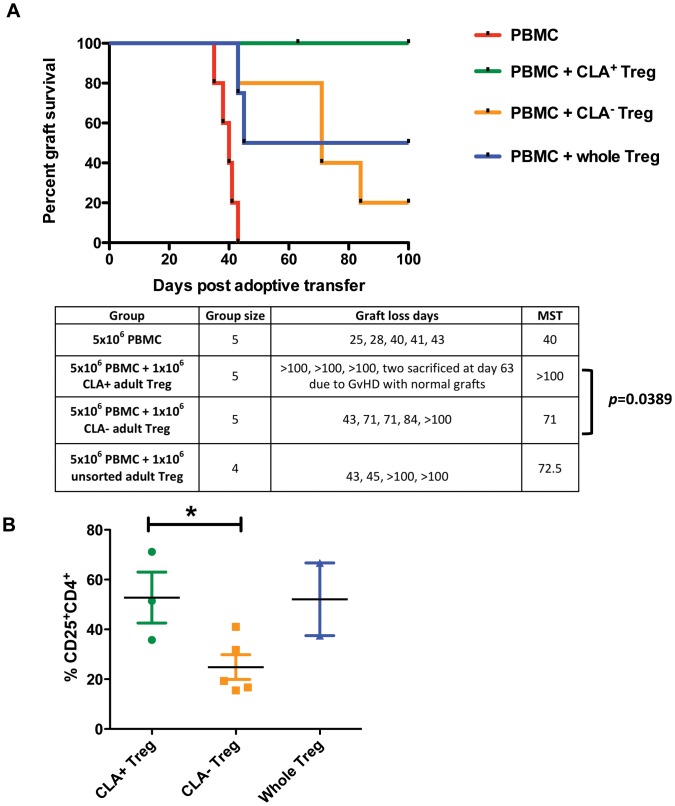
Skin-homing *CLA^+^ Treg are more effective at preventing allograft destruction than CLA* ^−^ *Treg.* (**A**) APB-derived Treg were sorted into CLA^+^ and CLA^−^ subpopulations and adoptively transferred together with non-autologous PBMC at a 5∶1 ratio of PBMC to Treg into mice previously transplanted with a human skin allograft. A further group of mice received unsorted APB-derived Treg. While CLA^−^ Treg treatment resulted in an MST of 71, treatment with CLA^+^ Treg significantly prolonged skin allograft survival to beyond 100 days (*p* = 0.0389). Mice receiving unsorted Treg achieved a similar MST to those receiving CLA^−^ Treg (72 days, *p* = 0.1827). (**B**) In mice treated with CLA^+^ Treg, a higher frequency of CD4^+^CD25^hi^ cells was detected within the graft on day 100 compared to mice treated with CLA^−^ Treg (*p* = 0.031).

As the presence of Treg within a skin graft is sufficient to prevent its alloimmune-mediated destruction (**Figure 1F and 1G**), we hypothesised that Treg capable of migrating to skin are more effective at suppressing alloresponses against skin *in vivo.* Having demonstrated a higher expression of the skin homing molecule CLA on APB-derived Treg and higher numbers of Treg within skin grafts of mice treated with APB-derived Treg, we investigated the hypothesis that CLA expressing APB-derived Treg will be more efficient at preventing human skin allograft destruction than their CLA^−^ counterparts. Human skin-transplanted BALB/c Rag2^−/−^cγ^−/−^ mice were reconstituted with allogeneic PBMC and non-autologous APB-derived Treg that were sorted based on their CLA expression, or whole unsorted Treg (all from the same donor). CLA^+^ Treg were effective at prolonging the survival of the skin transplant, achieving an MST of >100 days compared with 71 days using CLA^−^ Treg (*p*<0.05) or 72.5 days using whole unsorted Treg (**Figure 4A**). Importantly, a higher frequency of CD4^+^CD25^hi^ cells within the skin allografts was detected in mice treated with CLA^+^ Treg as compared with CLA^−^ Treg (**Figure 4B**).

In summary, we have demonstrated that the ability of Treg to home to skin is important in determining their efficacy in preventing alloimmune-mediated damage. Treg populations that do not express the necessary tissue-homing molecules are therefore less effective at preventing skin pathology *in vivo* despite exhibiting similar levels of *in vitro* suppressive activity. Duhen and colleagues recently phenotyped human Treg according to the expression of a number of chemokine receptors, identifying four separable populations of FOXP3^+^ cells with similar *in vitro* suppressive activity [10]. One of these populations displayed a phenotype that mirrored Th22 cells (‘Th22-like Treg’), expressing high levels of CLA [20], [21]. We propose that preparations of APB-derived Treg contain a significant proportion of ‘Th22-like’ CLA expressing Treg, and are thus capable of efficiently regulating skin inflammatory responses. The migratory capabilities of Treg are important in the prevention of autoimmune as well as alloimmune pathologies: the chemokine-mediated recruitment of Treg into the site of an immune response is also important for the prevention of autoimmune diabetes [11]. Treg that express skin-homing molecules may therefore also be useful for the treatment of autoimmune skin pathology [13]. The expression of lymphoid-specific homing receptors is also important, as Treg act in the allograft-draining lymph nodes to prevent the priming of effector T cells [14], [22], [23]. However whether lymphoid-homing alone is sufficient for the regulation of an immune response is unclear. In an elegant mouse study by Tomura and colleagues, Treg were shown to traffic between the skin and its draining lymph nodes. Importantly, skin-emigrating Treg were more effective at inhibiting cutaneous immune responses than lymph node-resident Treg [24], suggesting that the ability of Treg to circulate between peripheral and lymphoid tissues is important for efficient regulation.

Numerous phenotypic markers allow the subdivision of Treg into discrete subpopulations. For example, the differential expression of CD45RA and CD45RO on Treg is important for determining the capacity for proliferation and suppression [2], [17]. Here we demonstrate that the expression of CD45RO is associated with CLA expression and is thus important in *in vivo* settings where the homing of Treg to skin is necessary, such in the treatment of GvHD-related skin pathology. Treg that are currently in development for therapeutic use in BMT are being derived from a variety of sources and isolated through a variety of techniques [6], [7]. Such diverse Treg populations, while displaying similar *in vitro* suppressive capabilities [10] may behave differently *in vivo*. It is therefore essential that a Treg population being selected as a clinical cellular therapy for a specific pathology expresses the necessary migratory characteristics to allow its migration into the tissue where regulation is required.

## Methods

### Ethics Statement

Experiments were performed using protocols approved by the Committee on Animal Care and Ethical Review at the University of Oxford and in accordance with the UK Animals (Scientific Procedures) Act 1986. For the collection of human tissue samples, this was performed with full informed written consent and with ethical approval from the Oxfordshire Research Ethics Committee (REC B), study number 07/H0605/130.

### Mice

BALB/c Rag2^−/−^cγ^−/−^ (H2d) mice were housed under specific pathogen-free conditions in the Biomedical Services Unit of the John Radcliffe Hospital (Oxford, UK).

### Animal Procedures

Skin transplantation was performed as previously described [16]. Only mice displaying >1% splenic human leukocyte chimerism were included in analyses.

### Isolation of Skin Infiltrating Leukocytes from Human Skin

The method of isolating skin infiltrating leukocytes was adapted from McLachlan et al. [25]. Briefly, skin was placed in ice-cold 2% FCS RPMI-1640, divided into small pieces and incubated with 1.6 mg/ml Collagenase D (Roche) at 37°C and 5% CO_2_ for 30 minutes. Skin fragments were then mashed with the back end of a syringe plunger and incubated for an additional 20–30 minutes. Skin pieces were then filtered though a 70 µn nylon mesh, mashed again and rinsed with a solution of PBS with 5 mM EDTA and 2% FCS. Collected cells were stained for flow cytometric analysis.

### Isolation and Expansion of Human CD127^lo^ Regulatory T cells

Fresh adult buffy coats and cord blood units were obtained from the National Blood Service (UK). Isolation and expansion of Treg was performed as previously described with minor modifications [16]. Adult CD127^lo^CD25^+^CD4^+^ cells were sorted from CD25^+^-enriched cells using a BD FACSAria cell sorter. Cord blood Treg isolation was performed using a CD25^+^ isolation kit (Miltenyi, UK). Adult Treg were expanded with 1000 U/ml of recombinant human (rh) IL-2 (Chiron) and αCD3/αCD28 beads (Invitrogen) in a 1∶3 (1^st^ round) and 1∶1 (2^nd^ round) cell to bead ratio over two 7-day rounds, followed by 2 days of silencing in a reduced amount of rhIL-2 (200U/ml) and αCD3/αCD28 bead removal. Cord blood Treg were expanded with 1000U/ml of rhIL-2 and αCD3/αCD28 beads in a 1∶3 cell to bead ratio over 14 days, followed by 2 days of silencing.

### In vitro Suppression Tests

Treg *in vitro* suppressive activity was assessed by measuring inhibition of proliferation of non-autologous PBMC stimulated with αCD3/αCD28 beads. CFSE-labelled PBMCs (5×10^4^) were incubated for 72 hours with αCD3/αCD28 beads (1×10^4^) in the presence of *in vitro* expanded Treg before analyses by flow cytometry. A division index was calculated in a similar manner to Roederer et al. [26].

### Flow Cytometry

Fluorochrome-coupled antibodies specific for CD4 (Beckman Coulter), CD45, CD8, CD19, CD25, CD27, CCR7, CLA, CD127 (all BD), FOXP3, CD3, 7-AAD (eBioscience) and CD62L (Invitrogen) were used to phenotypically profile cells. Data were acquired using a FACSCanto and analysed using FACSDiva software (BD).

### Tissue Typing

Donor blood was analysed at the Oxford Transplant Centre Histocompatibility Laboratory for HLA-A, -B, -Cw, -DR and –DQ haplotypes.

### Immunohistochemistry

Immunohistochemistry was performed as previously described [16] **.** Snap-frozen specimens were sectioned at 8µm and stained using mouse anti-human FOXP3 antibody (236/A, a gift from Professor Alison Banham) and haematoxylin counterstaining. For infiltrating cell quantification, positive cells were counted at 40× magnification in three random fields of each of four separate sections per sample.

### Statistical Analyses

Student’s *t* tests were applied on grouped data. Survival data were analysed using logrank tests.

## References

[1] Godfrey WR, Spoden DJ, Ge YG, Baker SR, Liu B, et al (2005) Cord blood CD4(+)CD25(+)-derived T regulatory cell lines express FoxP3 protein and manifest potent suppressor function. Blood 105: 750–758. 15374887 10.1182/blood-2004-06-2467

[2] Miyara M, Yoshioka Y, Kitoh A, Shima T, Wing K, et al (2009) Functional delineation and differentiation dynamics of human CD4+ T cells expressing the FoxP3 transcription factor. Immunity 30: 899–911. 19464196 10.1016/j.immuni.2009.03.019

[3] Santner-Nanan B, Seddiki N, Zhu E, Quent V, Kelleher A, et al (2008) Accelerated age-dependent transition of human regulatory T cells to effector memory phenotype. Int Immunol 20: 375–383. 18195049 10.1093/intimm/dxm151

[4] Wood KJ, Bushell A, Jones ND (2011) Immunologic unresponsiveness to alloantigen in vivo: a role for regulatory T cells. Immunol Rev 241: 119–132. 21488894 10.1111/j.1600-065X.2011.01013.x

[5] McMurchy AN, Bushell A, Levings MK, Wood KJ (2011) Moving to tolerance: Clinical application of T regulatory cells. Semin Immunol. 10.1016/j.smim.2011.04.001 PMC3836227 21620722

[6] Brunstein CG, Miller JS, Cao Q, McKenna DH, Hippen KL, et al (2011) Infusion of ex vivo expanded T regulatory cells in adults transplanted with umbilical cord blood: safety profile and detection kinetics. Blood 117: 1061–1070. 20952687 10.1182/blood-2010-07-293795 PMC3035067

[7] Di Ianni M, Falzetti F, Carotti A, Terenzi A, Castellino F, et al (2011) Tregs prevent GVHD and promote immune reconstitution in HLA-haploidentical transplantation. Blood 117: 3921–3928. 21292771 10.1182/blood-2010-10-311894

[8] Tan MC, Goedegebuure PS, Belt BA, Flaherty B, Sankpal N, et al (2009) Disruption of CCR5-dependent homing of regulatory T cells inhibits tumor growth in a murine model of pancreatic cancer. J Immunol 182: 1746–1755. 19155524 10.4049/jimmunol.182.3.1746 PMC3738070

[9] Hirahara K, Liu L, Clark RA, Yamanaka K, Fuhlbrigge RC, et al (2006) The majority of human peripheral blood CD4+CD25highFoxp3+ regulatory T cells bear functional skin-homing receptors. J Immunol 177: 4488–4494. 16982885 10.4049/jimmunol.177.7.4488

[10] Duhen T, Duhen R, Lanzavecchia A, Sallusto F, Campbell DJ (2012) Functionally distinct subsets of human FOXP3+ Treg cells that phenotypically mirror effector TH cells. Blood. 10.1182/blood-2011-11-392324 PMC3362361 22438251

[11] Montane J, Bischoff L, Soukhatcheva G, Dai DL, Hardenberg G, et al (2011) Prevention of murine autoimmune diabetes by CCL22-mediated Treg recruitment to the pancreatic islets. J Clin Invest 121: 3024–3028. 21737880 10.1172/JCI43048 PMC3148722

[12] Ding Y, Xu J, Bromberg JS (2012) Regulatory T cell migration during an immune response. Trends Immunol 33: 174–180. 22305714 10.1016/j.it.2012.01.002 PMC3319498

[13] Klarquist J, Denman CJ, Hernandez C, Wainwright DJ, Strickland FM, et al (2010) Reduced skin homing by functional Treg in vitiligo. Pigment Cell Melanoma Res 23: 276–286. 20175879 10.1111/j.1755-148X.2010.00688.x PMC3778930

[14] Carvalho-Gaspar M, Jones ND, Luo S, Martin L, Brook MO, et al (2008) Location and time-dependent control of rejection by regulatory T cells culminates in a failure to generate memory T cells. J Immunol 180: 6640–6648. 18453583 10.4049/jimmunol.180.10.6640

[15] Graca L, Cobbold SP, Waldmann H (2002) Identification of regulatory T cells in tolerated allografts. J Exp Med 195: 1641–1646. 12070291 10.1084/jem.20012097 PMC2193557

[16] Issa F, Hester J, Goto R, Nadig SN, Goodacre TE, et al (2010) Ex vivo-expanded human regulatory T cells prevent the rejection of skin allografts in a humanized mouse model. Transplantation 90: 1321–1327. 21048528 10.1097/TP.0b013e3181ff8772 PMC3672995

[17] Booth NJ, McQuaid AJ, Sobande T, Kissane S, Agius E, et al (2010) Different proliferative potential and migratory characteristics of human CD4+ regulatory T cells that express either CD45RA or CD45RO. J Immunol 184: 4317–4326. 20231690 10.4049/jimmunol.0903781

[18] Clark RA, Chong B, Mirchandani N, Brinster NK, Yamanaka K, et al (2006) The vast majority of CLA+ T cells are resident in normal skin. J Immunol 176: 4431–4439. 16547281 10.4049/jimmunol.176.7.4431

[19] Josefowicz SZ, Rudensky A (2009) Control of regulatory T cell lineage commitment and maintenance. Immunity 30: 616–625. 19464984 10.1016/j.immuni.2009.04.009 PMC4410181

[20] Duhen T, Geiger R, Jarrossay D, Lanzavecchia A, Sallusto F (2009) Production of interleukin 22 but not interleukin 17 by a subset of human skin-homing memory T cells. Nat Immunol 10: 857–863. 19578369 10.1038/ni.1767

[21] Trifari S, Kaplan CD, Tran EH, Crellin NK, Spits H (2009) Identification of a human helper T cell population that has abundant production of interleukin 22 and is distinct from Th-17, Th1 and Th2 cells. Nat Immunol 10: 864–871. 19578368 10.1038/ni.1770

[22] Ermann J, Hoffmann P, Edinger M, Dutt S, Blankenberg FG, et al (2005) Only the CD62L+ subpopulation of CD4+CD25+ regulatory T cells protects from lethal acute GVHD. Blood 105: 2220–2226. 15546950 10.1182/blood-2004-05-2044

[23] Oliveira V, Sawitzki B, Chapman S, Appelt C, Gebuhr I, et al (2008) Anti-CD4-mediated selection of Treg in vitro - in vitro suppression does not predict in vivo capacity to prevent graft rejection. Eur J Immunol 38: 1677–1688. 18465768 10.1002/eji.200737562 PMC2988420

[24] Tomura M, Honda T, Tanizaki H, Otsuka A, Egawa G, et al (2010) Activated regulatory T cells are the major T cell type emigrating from the skin during a cutaneous immune response in mice. J Clin Invest 120: 883–893. 20179354 10.1172/JCI40926 PMC2827959

[25] McLachlan JB, Catron DM, Moon JJ, Jenkins MK (2009) Dendritic cell antigen presentation drives simultaneous cytokine production by effector and regulatory T cells in inflamed skin. Immunity 30: 277–288. 19200757 10.1016/j.immuni.2008.11.013 PMC2770245

[26] Roederer M (2011) Interpretation of cellular proliferation data: avoid the panglossian. Cytometry Part A : the journal of the International Society for Analytical Cytology 79: 95–101. 21265003 10.1002/cyto.a.21010

